# MiR-337-3p Promotes Adipocyte Browning by Inhibiting TWIST1

**DOI:** 10.3390/cells9041056

**Published:** 2020-04-23

**Authors:** Indira G.C. Vonhögen, Hamid el Azzouzi, Servé Olieslagers, Aliaksei Vasilevich, Jan de Boer, Francisco J. Tinahones, Paula A. da Costa Martins, Leon J. de Windt, Mora Murri

**Affiliations:** 1 Department of Molecular Genetics, Faculty of Sciences and Engineering, CARIM School for Cardiovascular Diseases, Faculty of Health, Medicine and Life Sciences, Maastricht University, 6200 MD Maastricht, The Netherlands; indira.vonhogen@maastrichtuniversity.nl (I.G.C.V.); h.elazzouzi@erasmusmc.nl (H.e.A.); s.olieslagers@maastrichtuniversity.nl (S.O.); p.dacostamartins@maastrichtuniversity.nl (P.A.d.C.M.); 2 Department of Molecular Genetics, Erasmus University MC, 3015 GD Rotterdam, The Netherlands; 3 BioInterface Science Group, Department of Biomedical Engineering and Institute for Complex Molecular Systems, Eindhoven University of Technology, 5600 MB Eindhoven, The Netherlands; a.vasilevich@tue.nl (A.V.); j.d.boer@tue.nl (J.d.B.); 4 Unidad de Gestión Clínica de Endocrinología y Nutrición, Instituto de Investigación Biomédica de Málaga (IBIMA), Hospital Clínico Virgen de la Victoria, 29010 Málaga, Spain; fjtinahones@hotmail.com (F.J.T.); moramurri@gmail.com (M.M.); 5 Centro de Investigación Biomédica en Red de Fisiopatología de la Obesidad y la Nutrición, CIBERObn, Instituto de Salud Carlos III, 28029 Madrid, Spain; 6 Faculty of Medicine, University of Malaga, 29010 Malaga, Spain; 7 Department of Physiology and Cardiothoracic Surgery, Faculty of Medicine, University of Porto, 4200-319 Porto, Portugal

**Keywords:** brown adipose tissue, microRNA, mitochondria, obesity, metabolic syndrome

## Abstract

The prevalence of metabolic syndrome (MetS) and obesity is an alarming health issue worldwide. Obesity is characterized by an excessive accumulation of white adipose tissue (WAT), and it is associated with diminished brown adipose tissue (BAT) activity. Twist1 acts as a negative feedback regulator of BAT metabolism. Therefore, targeting Twist1 could become a strategy for obesity and metabolic disease. Here, we have identified miR-337-3p as an upstream regulator of Twist1. Increased miR-337-3p expression paralleled decreased expression of TWIST1 in BAT compared to WAT. Overexpression of miR-337-3p in brown pre-adipocytes provoked a reduction in Twist1 expression that was accompanied by increased expression of brown/mitochondrial markers. Luciferase assays confirmed an interaction between the miR-337 seed sequence and *Twist1* 3′UTR. The inverse relationship between the expression of *TWIST1* and miR-337 was finally validated in adipose tissue samples from non-MetS and MetS subjects that demonstrated a dysregulation of the miR-337-Twist1 molecular axis in MetS. The present study demonstrates that adipocyte miR-337-3p suppresses Twist1 repression and enhances the browning of adipocytes.

## 1. Introduction

The worldwide prevalence of obesity has nearly tripled since 1975; more than 2.1 billion people are affected by obesity and overweight. It is a major health problem as it is associated with an increased risk of morbidity and mortality in the world, causing 3.4 million deaths and 3.9% of years of life lost worldwide [1]. Overall, obesity is an insidious condition that is often complicated by the co-occurrence of hyperglycaemia, dyslipidaemia, and hypertension, which are classified together as the metabolic syndrome (MetS) [2]. Therefore, obesity requires effective therapeutic interventions. A proportion of obese individuals are protected against worsening of metabolic health, whereas, at the other end of the spectrum, there are normal weight individuals who have metabolic abnormalities usually associated with obesity, suggesting that adipose tissue physiology, rather than the amount of fat mass, may be a key factor in the pathophysiology of obesity-related metabolic disorders [3,4].

In recent years, adipose tissue has been recognized as a major endocrine organ, which plays a key role in energy homeostasis. In mammals, two types of adipose tissue can be distinguished from a histological and functional perspective: white (WAT) and brown adipose tissue (BAT) [5]. WAT is mainly involved in lipid storage within lipid droplets in the fed state, and release of fatty acids from the breakdown of stored triglycerides in the fasted state, whereas BAT utilizes neutral lipids stored in lipid droplets for the generation of heat and to meet cellular energetic requirements [5,6]. Morphologically, WAT adipocytes contain a single lipid droplet, and few mitochondria, while BAT adipocytes contain a high number of mitochondria and multiple small lipid droplets. In the last few years, beige adipocytes have been reported in WAT, which are brown-like adipocytes that contain medium mitochondria density and a few to many lipid droplets, and also have thermogenic capacity [7]. Obesity is associated with lower brown/beige adipocyte activity. A recent study has shown that obesity led to the development of a hypoxic state in BAT, diminished β-adrenergic signaling, mitochondrial dysfunction and loss, causing obesity-associated BAT whitening [8].

The essential role of mitochondria in numerous aspects of metabolic regulation, including energy supply, regulation of apoptosis, calcium homeostasis, and production of reactive oxygen species, position them at the center of the control of global energy homeostasis [9,10]. Mitochondrial metabolism is both the origin and target of multiple nutrient signals that orchestrate integrated physiological responses to maintain cellular insulin-sensitivity. To perform their key roles in cellular energy production, mitochondria use an intricate system that encompasses the breakdown of fatty acids and glucose, which is coupled to oxidative phosphorylation, generating cellular energy in the form of adenosine triphosphate (ATP). This central position of mitochondria makes them vulnerable to damage [11]. A metabolic imbalance of nutrient supply, energy production, and/or oxidative respiration results in “mitochondrial dysfunction”.

MicroRNAs (miRs) are functional non-coding RNAs that regulate gene expression at the post-transcriptional level. They play an important regulatory role in a variety of biological processes, including regulators of differentiation, development, and function of brown and beige adipocytes. Recently, several studies have identified several miRs involved in the regulation of browning [12,13]. In terms of therapeutic potential, miRs represent a novel and an attractive target to manipulate malignant body functions as their activity can be efficiently modulated with RNA-based antisense technology [14,15]. A transcriptional regulator, Twist1, has been reported to be mainly present in the adipose tissue and acts as a negative regulator of brown fat metabolism [16]. An in vivo study showed that transgenic mice expressing twist-1 in the adipose tissue are susceptible to high-fat diet-induced obesity, whereas twist-1 heterozygous knockout mice showed obesity-resistance [16]. Therefore, targeting Twist1 by miRNA intervention could become a strategy for the treatment of obesity and metabolic disease.

The aim of the present study was to identify a miR that targets Twist1 to modulate browning of adipocytes. A bioinformatic screen resulted in the identification of potential miRs capable of repressing Twist1 in adipocytes, promoting browning by counteracting the inhibition of brown fat metabolism and mitochondrial biogenesis. As a validation of this screen, miR-337-3p was found to be most enriched in BAT. Overexpression of miR-337-3p enhanced brown fat metabolism by a reduction in Twist1 and an increased expression of the downstream uncoupling protein 1 (UCP1) thermoregulatory effector protein. The proposed relationship was further substantiated by validation of seed complementarity, confirming the interaction between miR-337-3p and Twist1 3′UTR. Altogether, our results show that miR-337-3p plays a role in the modulation of browning of adipocytes by targeting Twist1. The concordance of the proposed mechanism and validation in human samples has raised interesting grounds for corroborating its therapeutic potential in counteracting obesity and metabolic syndrome.

## 2. Materials and Methods

### 2.1. Screening of Brown Inducing miR Candidates

Genes repressing browning of adipose tissue were selected based on a literature screen performed in PUBMED (Table S2). miR candidates were identified by imputing selected genes identified through literature in miR target prediction tools, miR-Base (http://www.mirbase.org/; The University of Manchester, Manchester, UK) and miRNAviewer (http://cbio.mskcc.org/mirnaviewer/; Memorial Sloan-Kettering Cancer Center, New York, NY USA) (Table S2). Candidates were further screened, analysing the endogenous expression in mouse brown and white adipose tissue by quantitative real-time PCR. Following the screen, the miRNA candidate that was most enriched in mouse BAT compared to WAT was further validated by in vitro functional analysis for its browning ability. Throughout our manuscript, we adhered to the animal research: reporting of in vivo experiments (ARRIVE) guidelines (https://www.nc3rs.org.uk/arrive-guidelines) for reporting the in vivo animal studies.

### 2.2. Quantitative RT-PCR

Total RNA was extracted from mouse and human tissue and mouse-derived cultured cell lines using TRIzol reagent from the Direct-Zol™ RNA Miniprep kit (R2051) following the manufacturer’s protocol. Total RNA was reversely transcribed using a miScript II RT Kit (218161 Qiagen, Hilden, Germany), while mRNA templates were reversely transcribed using the Moloney Murine Leukemia Virus (M-MLV RT) reverse transcriptase by Promega (9PIM170; Promega, Madison, WI, USA). RT-PCR was performed using SYBR green, carried out on a Bio-Rad CFX96 Real-Time System. For validation of transfection efficiency in cell culture experiments, miR-337-3p pre-designed miRCURY LNA Uni RT primer mix (339306, Qiagen) was used. Consequently, total RNA was reversely transcribed using its compatible miRCURY LNA™ Universal cDNA synthesis kit II (203301, Qiagen) followed by real-time PCR with the LNA enhanced primers using ExiLENT SYBR green (203401, Thermofisher, Waltham, MA, USA). Quantification of transcript level by RT-PCR was done by using the relative Ct (ΔΔCt) method. mRNA transcripts were normalized to *L7*, while miR transcripts were normalized to *U6* or *5s* (pre-designed miRCURY LNA Uni RT primer mix, Qiagen). The choice of reference genes was based on their stability in the different samples analyzed. The sequence of the primers used in this study can be found in Table 1.

### 2.3. Western Blot Analysis 

Whole cell and tissue lysates were homogenized in protein lysis buffer (IGEPAL 1%, SDS 0.5%, NaCl 150 nM, Tris-HCl 50 mM pH 8.5, EDTA 5 mM) supplemented by a protease inhibitor cocktail and PhosSTOP complete (Roche Applied Sciences, Penzberg, Bavaria, Germany). Supernatants from cell lysates were briefly centrifuged, while tissue lysates were centrifuged successively (12,000× *g*, 4 °C, 20 min) followed by protein quantification by the BCA Protein Assay Kit (Thermo Scientific, Rockford, IL, USA). Samples supplemented by 4× Laemmli buffer and 5% β-mercaptoethanol were boiled at 42 °C for 25 min, separated by 10% and 12% SDS-polyacrylamide gel and transferred to nitrocellulose blotting membrane (Amersham™ Protran™ 0.45 µM NC, GE Healthcare LifeScience, Chicago, Il, USA). Blotted membranes from tissue samples were stained for total protein detection using the Li-COR REVERT Kit following the manufacturer’s instructions (Li-Cor Biosciences GmbH, Bad Homburg vor der Höhe, Germany. Total protein stain (TPS) was then removed by reversal solution LI-COR 0.1M sodium Hydroxide, 30% methanol in water. Next, membranes were blocked for 1 h at room temperature in Odyssey PBS blocking buffer (LI-COR 927-40100), and each membrane was incubated with primary antibodies in Odyssey PBS blocking buffer 0.1% Tween) overnight at 4 °C. Next, membranes were washed 3× (PBS-T), incubated at room temperature for 1 h with secondary antibodies, washed 3× PBS-T, and imaged (multiplexed analysis) on an Odyssey Clx imaging system (LI-COR) at an automatic scanning resolution of 169 µm and automatic image quality setting. Images were analyzed by quantification of band densitometry by ImageJ. For single target detection, membranes were blocked with 10% milk in TBS-T, all washing and incubations were performed in TBS-T 5% milk and incubations with secondary antibodies followed by image detection. Furthermore, images for single target detection were generated using Extreme Sensitivity Chemiluminescence Substrate (NEL 112001EA) on a 3000 image Analyzer (Fujifilm). Primary antibodies included anti-TWIST1 (T6451 Sigma affinity isolated produced in rabbit 1:500; Sigma-Aldrich, Saint Louis, MO, USA); OXPHOS rodent antibody cocktail (Abcam 110413 1:1000, Abcam, Cambridge, UK); anti-UCP1 (U6382 Sigma produced in rabbit 1:1000); anti-ACTB (A1978 Sigma monoclonal produced in mice 1:1000) as the loading control for cell lysates and anti-GAPDH (1:1000) Mouse Monoclonal (Millipore MAB374, Merck-Millipore, Burlington, MA, USA) as the loading control for tissue lysates. Secondary antibodies included IRDye 800CW Goat anti-Mouse IgG (LI-COR 926 32210) and IRDye 680RD Donkey anti-Rabbit (LI-COR 925 68073) at 1:10000 for multiple target detection, and polyclonal rabbit anti-mouse IgG-HRP (DAKO 1:2000, Agilent Technologies, Santa Clara, CA, USA) and polyclonal swine anti-rabbit IgG-HRP (DAKO 1:2000, Agilent Technologies) for single target detection. Results are presented in densitometry arbitrary units as an n-fold increase over ACTB for cells, GAPDH, or TPS for tissue samples.

### 2.4. Cell Culture

Immortalized brown pre-adipocytes were obtained from Dr. Bruce Spiegelman, Harvard Medical Center, and maintained in DMEM (1×)-Glutamax (Gibco) with 20% FBS and 100 µg/mL Pen/Strep (Gibco-BRL, Life Technologies Ltd., Paisly, UK) in a humidified atmosphere of 5% CO_2_ at 37 °C [17]. Immortalized brown pre-adipocytes were reversely transfected with 10 nM of precursor or 100 nM of miRCURY LNA inhibitor specific for mmu-miR-337-3p or precursor scrambled miR or LNA inhibitor negative control, respectively, with 4 µL of Oligofectamin (Invitrogen, Life Technologies, Carlsbad, CA, USA) per well of a 6-wells plate following the manufacturer’s protocol. Transfected pre-adipocytes were grown to confluence (day 0) and induced to differentiation as previously described [17].

### 2.5. Luciferase Assay

Constructs bearing 713 base pairs of murine *Twist1* 3′ untranslated region (UTR) were sub-cloned in pmiRGLo Dual-Luciferase miR Target expression Vector (Promega, Madison, WI, USA). COS7 cells maintained in DMEM, with 10% FBS, 100 µg/mL Penicillin/Streptomycin, 10 µg/mL gentamycin, 100 µg/mL l-glutamine, and seeded in 48-well plates (24,000 cells/well) were transfected with the reporter plasmid containing Twist-1 3′ untranslated region (UTR) using X-tremeGene 9 DNA transfection reagent followed by co-transfection with mmu-miR-337-3p precursor or LNA inhibitor or precursor scrambled miR negative control or LNA inhibitor negative control using oligofectamin transfection reagent (Invitrogen), as previously described. After 48 h, the luciferase activity was quantified using a Dual-Glo luciferase assay system (Promega) on a Luminometer. Firefly luciferase activity was normalized to renilla luciferase activity as an internal control.

### 2.6. Immunofluorescence and Detection of Mitochondrial Activity

Immortalized brown pre-adipocytes were grown to confluence (day 0) and induced to differentiation as previously described [17]. At day 0 and day 5 of differentiation, cells were washed twice with PBS and fixed with 4% paraformaldehyde. Mitochondrial staining of day 0 and day 5 cells was performed by mouse monoclonal Anti-ATPB Antibody (Abcam ab14730 1:500, Abcam) with overnight incubation at room temperature, washed 2 times with PBS followed by Secondary Goat anti-Mouse IgG H&L (Abcam ab150119 1:5000, Abcam) and HSC LipidTOX™ Green neutral lipid stain (Invitrogen H34475 1:1000, Life Technologies) for lipid droplet staining, then incubated for 1 h at room temperature. Subsequently, cells were washed 2 times with PBS and stained with DAPI (Sigma D9542 1:2000, Sigma-Aldrich) and imaged with a Nikon (Tokyo, Japan) Eclipse Ti_E epi-fluorescent inverted microscope. The sample was excited with an LED Spectra4 light source either with 390, 475, 549, or 632 nm. The emitted light, with wavelength 430, 488, 561, or 647 nm, correspondingly, were recorded with an Andor Zyla 5.5 4MP Mono camera. Number, integrated staining intensity, and area of mitochondria and lipid droplets were quantified from day 0 and day 5 cells and corrected for the total number of cells per image and displayed as median ± SD.

### 2.7. Mouse Adipose Tissue Isolation

Mouse WAT was isolated from inguinal subcutaneous adipose fat depots, while BAT was isolated from the interscapular brown adipose fat depot, from wild type adult mouse in a BL6BAF1 background (n = 8). After extraction of fat depots, the tissue was immediately frozen by immersion in liquid nitrogen and stored at −80 °C for subsequent RNA and protein extraction. Identity of BAT and WAT was confirmed by RNA expression of BAT specific markers. The mice used in the study were housed in climate-controlled, 12 h light–dark cycle with ad libitum access to chow diet and water. Experimental procedures involving animals were reviewed and approved by the Animal Facility of Maastricht University (DEC2014-076 on the 13th of May 2016).

### 2.8. Human Sample Collection

Subcutaneous and visceral adipose tissue (SAT and VAT) was obtained from MetS subjects submitted to bariatric surgery or non-MetS subjects submitted to laparoscopic surgery due to hiatal hernia or cholelithiasis [18]. The patients completed a structured interview to obtain the following data: sex, age, medical history, and drug consumption. All subjects underwent a standardized anthropometric examination: weight, height, blood pressure, waist and hip circumferences, and biochemical parameters (Supplementary Table S1). None of the subjects were receiving orally administered antidiabetic agents or insulin therapy. Exclusion criteria included severe cardiac disease with prohibitive anaesthetic risks, major cardiovascular disease within 6 months prior to study inclusion, evidence of acute or chronic inflammatory disease, severe coagulopathy, tobacco and alcohol abuse, or inability to comply with nutritional requirements, including life-long vitamin replacement. All participants gave their informed consent, and the study was reviewed and approved on the 28th of December of 2015 by the ethics and research committee of Virgen de la Victoria Clinical University Hospital (Malaga, Spain).

### 2.9. Statistics

All results are displayed by means and standard error of means (SEM). Statistical analyses were performed in Prism (GraphPad Software, San Diego, CA, USA) and SPSS IBM 24 (IBM, Armonk, NY, USA). Normality of the distribution of continuous variables was assessed using the Shapiro–Wilk test. Logarithmic transformations ensured normal distribution of variables as needed. The relation between mouse WAT versus BAT; mouse BAT cells at day 0 vs day 5, BAT cells with precursor scramble vs. pre-miR-337-3p; BAT cells with LNA scramble vs. LNA-miR-337-3p; human non-MetS vs. MetS; were evaluated by either Mann–Whitney-U test for independent samples containing non-normal distributions or independent sample *t*-test for normal distribution data. For dependent samples, comparison between human SAT versus VAT, a Wilcoxon rank sum test was applied for non-normal distributions and Paired sample *t*-test for normal distributions. Spearman rank correlation analyses were performed for non-normal distributed data to analyze the correlation between miR expression levels from human adipose tissue and anthropometric and biochemical parameters. Significance level was set at *p* < 0.05. The heatmap was generated using Cluster 3.0, Mac OS X v10.0 (Stanford University, San Francisco, CA, USA) and Treeview software, V2.0.8 (Softonic International, Barcelona, Spain).

## 3. Results

### 3.1. miR-337 is a Potential Regulator of Browning of Adipose Tissue

**Figure 1:**
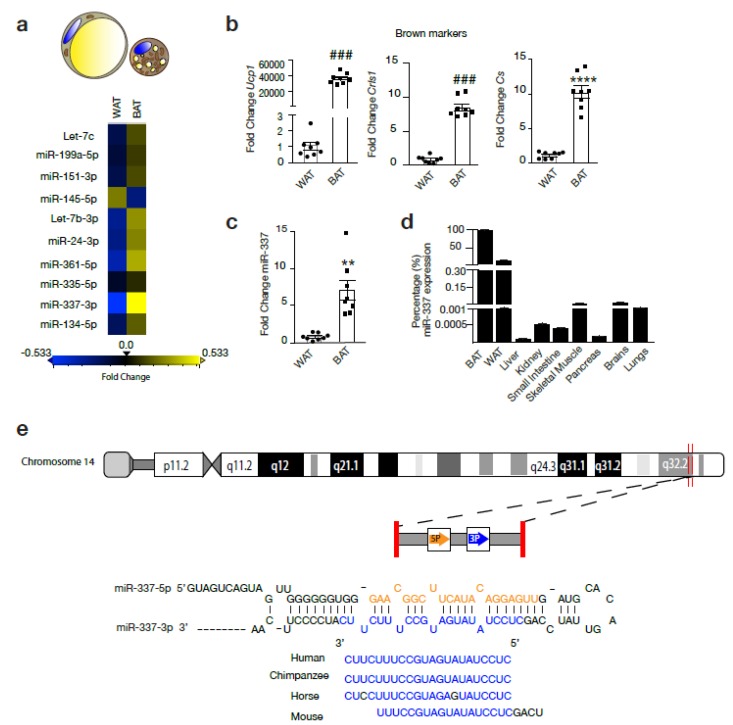
miR-337-3p is a potential regulator of browning of adipose tissue. (**a**) Representative heatmap showing the results of RT-PCR analysis used to screen a panel of microRNAs (miR) in mouse white adipose tissue (WAT) and brown adipose tissue (BAT): Let-7c, miR-199a-5p, miR-151-3p, miR-145-5p, Let-7b-3p, miR-24-3p, miR-361-5p, miR-335-5p, miR-337-3p, miR-134-5p. Real-time PCR was performed using the miRCURY LNA miR PCR Assay and normalized to *U6* RNA and relative to WAT. Differentially expressed miR (fold change ≥0.5) are indicated in yellow, which represents upregulated miRNA in BAT compared to WAT, and in blue are the downregulated miRs (fold change ≤−0.5) in BAT compared to WAT. N = 3/group. (**b**) RT-PCR analysis of transcript abundance of BAT thermogenic and mitochondrial markers *Ucp1*, *Cs*, and *Crls1* in BAT compared to WAT. mRNA expression values were normalized to *L7* mRNA. N = 8/group. (**c**) RT-PCR analysis of mmu-miR-337-3p expression in mouse WAT and BAT normalized to U6 RNA. N = 8/group (**d**) RT-PCR analysis of mmu-miR-337-3p abundance in BAT, WAT, liver, kidney, small intestine, skeletal muscle, pancreas, brains, and lungs presented as percentage of mmu-miR-337-3p transcript abundance from its expression in BAT. Expression of miR-337-3p in BAT is set to 100%. (**e**) Representative image of the genomic mapping of miR-337-3p in humans and conservation of its seed sequence among species. Data are presented as means ± SEM; individual data points are given by the dot plot. Either Mann–Whitney-U or Independent sample *t*-test was used to assess differences in expression levels. ### *p* < 0.001 Mann–Whitney-U, ** *p* < 0.01 **** *p* < 0.0001 Independent sample *t*-test, equal variances not assumed. n, number of biological replicas; WAT, white adipose tissue; BAT, brown adipose tissue; *Ucp1*, uncoupling protein-1; *Crls1*, cardiolipin synthase 1; *Cs*, Citrate synthase; RT-PCR, quantitative real time-polymerase chain reaction.

The expression of Let-7c, miR-199a-5p, miR-151-3p, miR-145-5p, Let-7b-3p, miR-24-3p, miR-361-5p, miR-337-3p, miR-134-5p were assessed in murine BAT and WAT. Among these candidate miRs, miR-337-3p showed the largest fold change with an increased expression in BAT compared to WAT. (Figure 1a–d) The brown expression marker and mitochondrial marker *Ucp1*, cardiolipin synthase (*Crls1*), and citrate synthase (*Cs*), respectively, confirmed BAT phenotype from adipose tissue isolated from the intrascapular region from mice (Figure 1b). miR-337-3p is an intergenic non-coding RNA located on chromosome 14 of the human genome overlapping with the Al 117190.1 long non-coding RNA, for which no particular function or interaction with miR-337-3p has been reported. Besides this, miR-337-3p is conserved among species indicating resistance to evolutionary pressure (Figure 1e). RT-PCR analysis revealed that although miR-337-3p was expressed in murine skeletal muscle, brain, and WAT with very low expression levels in liver, kidney, small intestine, and pancreas, it was most enriched in BAT (Figure 1d). In comparison to WAT, there is a 7-fold increase in the expression of miR-337-3p in BAT (Figure 1c).

### 3.2. miR-337 Is Upregulated During Browning

**Figure 2:**
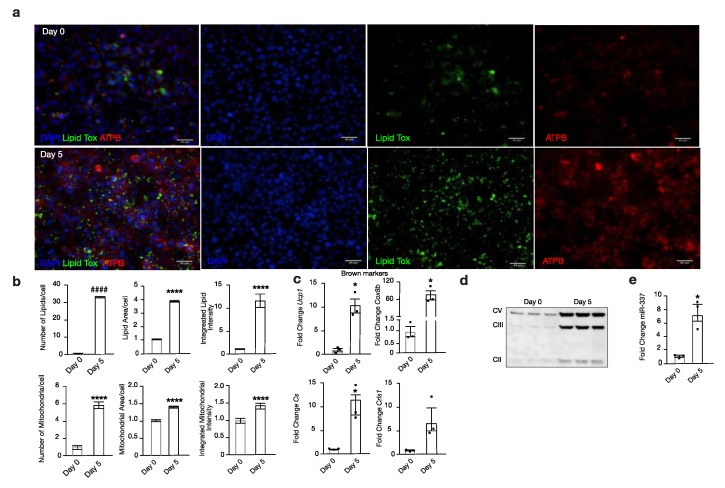
miR-337-3p is upregulated in browning. (**a**) Representative microscopic images of immunofluorescent stainings from day 0 and day 5 of mouse brown adipocyte differentiation for mitochondria (red) ATPB, lipid droplets (green) Lipidtox, and nuclear (blue) DAPI. The scale bar in the figures corresponds to 40µm. (**b**) Quantifications of number, integrated intensity, and area of mitochondria and lipids corrected per cell of day 0 and day 5 of mouse brown adipocyte differentiation. (n = 6 per group) (**c**) RT-PCR analysis of BAT thermogenic and mitochondrial markers, *Ucp1*, *Cox8b*, *Cs*, and *Crls1* of day 0 and day 5 of mouse brown adipocyte differentiation. L7 mRNA was used as an internal normalizer to the number of mRNA transcript copies. n = 3 per group. (**d**) Western blot analysis of Complex V (ATP5A-55kDa), III (UQCRC2-48 kDa), and II (SDHB-30kDa) of the electron transport chain of OXPHOS in day 0 and day 5 of mouse brown adipocyte differentiation. (**e**) RT-PCR analysis of mmu-miR-337-3p in day 0 and day 5 of mouse brown adipocyte differentiation, normalized to U6 RNA. N = 3/group. All immunofluorescent quantifications are presented as medians ± SD, while quantifications of RT-PCR analysis are presented as means ± SEM, and individual data points are given by the dot plot. An Independent sample *t*-test was used to assess differences in expression levels. * *p* < 0.05; **** *p* < 0.0001 by Independent sample *t*-test, equal variances not assumed. #### *p* < 0.0001 Independent sample *t*-test equal variances assumed. n, number of biological replicas; ATPB, β-subunit of ATP synthase; *Ucp1*, uncoupling protein-1; OXPHOS, oxidative phosphorylation; *Cox8b*, cytochrome C oxidase subunit 8b; *Cs*, citrate synthase; *Crls1*, cardiolipin synthase 1.

Next, we analysed miR-337-3p expression during the differentiation of murine brown pre-adipocytes (day 0) towards mature brown adipocytes (day 5). Successful differentiation of brown adipocytes was confirmed by an increased expression of *Ucp1*, cytochrome C oxidase subunit 8b (*Cox8b*), and *Cs*, whereas *Crls1* showed the same trend (Figure 2c). Phenotypical characterization by immunofluorescence showed an increase in both mitochondria (ATPB) and lipid droplets (Lipidtox™) from day 0 to day 5 of differentiation (Figure 2a,b). Western blot analysis confirmed an increased protein abundance of several complexes of OXPHOS, suggesting increased mitochondrial respiration from day 0 to day 5 of differentiation of brown adipocytes (Figure 2d). Ultimately, alongside these prominent changes occurring during the maturation of murine brown adipocytes, there is a 7-fold increased expression of miR-337-3p from day 0 to day 5 of differentiation (Figure 2e). Taken together, these findings indicate a potential role for miR-337-3p as a regulator of browning of adipose tissue and a possible modulator of mitochondrial function.

### 3.3. miR-337 Targets Twist1, a Negative Feedback Regulator of Brown Fat Metabolism

**Figure 3:**
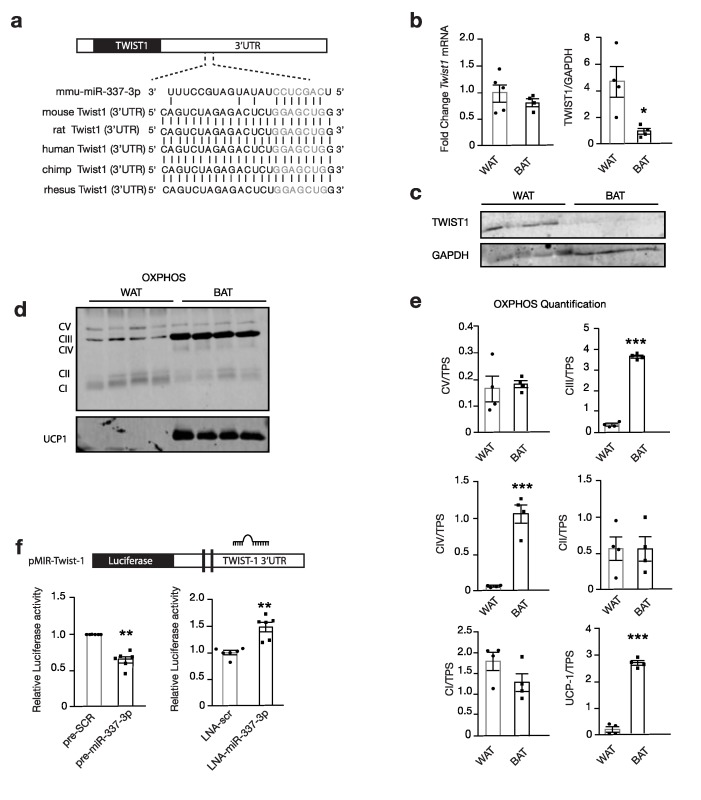
miR-337-3p targets Twist1. (**a**) Schematic representation of predicted seed complementarity between the seed sequence of mmu-miR-337-3p, and seed region on *Twist1* 3′UTR and the evolutionary conservation of this region among species. (**b**) RT-PCR and Western blot analysis of *Twist1* mRNA expression normalized to 5S (WAT n = 5, BAT n = 4) and TWIST1 protein abundance (WAT n = 4, and BAT n = 5) in mouse WAT and BAT normalized to GAPDH. (**c**) Representative Western blot of TWIST1 and GAPDH housekeeping protein in mouse WAT and BAT quantified in (**b**). (**d**) Western blot of mitochondrial OXPHOS, Complex V, IV, III, II, and I and thermogenic UCP1 protein. (**e**) Quantification of (**d**) OXPHOS and UCP1 protein abundance in mouse WAT and BAT normalized to TPS as the loading control. (**f**) Schematic of luciferase reporter plasmid containing Twist’1 3′UTR with a binding site for miR-337-3p. Relative luciferase activity quantification in Cos7 cells containing reporter plasmids, co-transfected with either mmu-miR-337-3p precursor or scrambled precursor, and co-transfected with LNA inhibitor for miR-337-3p or LNA inhibitor negative control. Data are presented as means ± SEM, and individual data points are given by the dot plot * *p* < 0.05; ** *p* < 0.01, by Independent sample *t*-test, equal variances not assumed. *** *p* < 0.001, by Independent sample *t*-test, equal variances assumed. n, number of biological replicas; 3′UTR, 3′ untranslated region; OXPHOS, oxidative phosphorylation; UCP1, uncoupling protein-1; GAPDH, Glyceraldehyde 3-phosphate dehydrogenase; *Twist1*, twist-related protein; RT-PCR, quantitative real-time polymerase chain reaction; TPS, total protein stain.

Twist1, a class I basic helix–loop–helix transcription factor and negative feedback regulator of *PPARGC1A*/PPARD-mediated brown fat metabolism, was identified as a propitious target for modulating brown fat metabolism. We hypothesized that miR-337-3p targets Twist1 and thereby, prevents the inhibition of brown fat metabolism (Figure 3a) [16]. Indeed, we found TWIST1 protein abundance to be significantly increased in murine WAT compared to BAT by Western blot analysis, and the inverse of the expression pattern of miR-337-3p (Figure 1c and Figure 3b–c). In line, the seed sequence of miR-337-3p shows perfect complementarity with *Twist1*-3′UTR and is evolutionarily conserved (Figure 3a). The expression of OXPHOS Complex III and Complex IV and UCP1 were increased in BAT compared to WAT, which is inverse to TWIST1 protein abundance (Figure 3c–e).

To confirm a physical interaction between miR-337-3p and the *Twist1*-3′UTR, luciferase reporters containing the responsive elements in the 3′UTR of *Twist1* with the hypothesized seed sequence were designed (Figure 3f). Co-transfection of reporter plasmids with precursor molecules for miR-337-3p in COS7 cells decreased luciferase reporter activity compared to transfection with the scrambled precursor, validating a physical interaction causing disruption of luciferase activity. Conversely, co-transfection with the miR inhibitor caused an increased luciferase activity compared to the scrambled inhibitor (Figure 3f). These findings indicate that miR-337-3p targets *Twist1* by interacting with its 3′ UTR.

### 3.4. Modulation of miR-337 Influences Twist1 and Modulates Mitochondrial Activity and Brown Fat Metabolism

**Figure 4:**
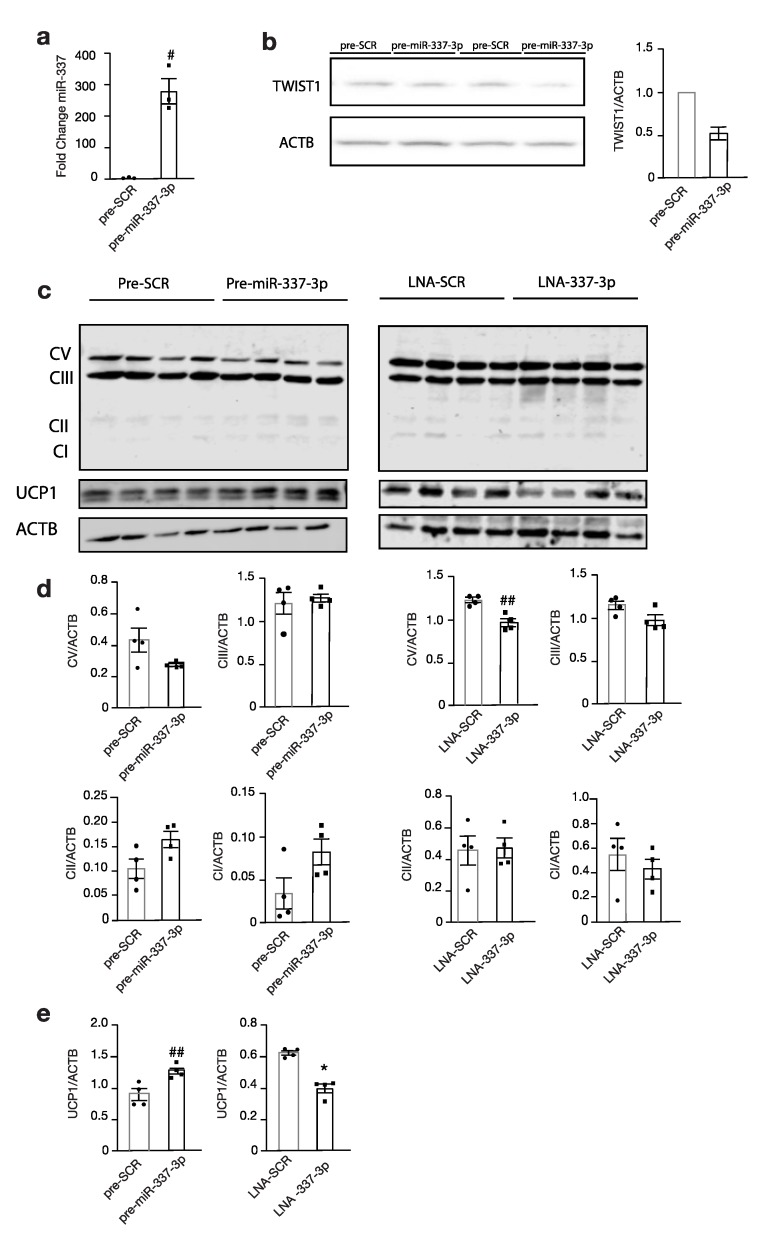
Modulation of miR-337-3p influences Twist1 and downstream thermogenic proteins. (**a**) RT-PCR analysis of mmu-miR-337-3p in murine brown adipocytes transfected with either precursor mmu-miR-337-3p or precursor scrambled molecule. (**b**) Western blot analysis of Twist-1 protein abundance of precursor mmu-miR-337-3p or precursor-scrambled transfected brown adipocytes and its quantification on the right (n = 2). (**c**) Western blot analysis of OXPHOS, Complex V-I, UCP1, and ACTB in brown adipocytes transfected with (left panel) either precursor mmu-miR-337-3p or precursor scrambled, or (right panel) transfected with LNA mmu-miR-337-3p inhibitor or scrambled inhibitor (n = 4 per group). (**d**) Quantification of OXPHOS Complex V-I protein abundance from (**c**) Western blot (**e**) Quantification of UCP1 protein abundance from (**c**) Western blot. Data are presented as means ± SEM, and individual data points are given by the dot plot. # *p* < 0.05, Independent sample *t*-test, equal variances not assumed * *p* < 0.05 Mann–Whitney-U for independent samples. ## *p* < 0.01, Independent sample *t*-test with equality of variances. n, number of biological replicas; OXPHOS, oxidative phosphorylation; UCP1, uncoupling protein 1; ACTB, beta-actin RT-PCR, quantitative real-time polymerase chain reaction.

Next, the expression of downstream targets of brown fat metabolism was investigated. Immortalized brown pre-adipocytes reversely transfected with precursor miR-337-3p (Figure 4a) showed a decrease in TWIST1 protein abundance compared to those transfected with the scrambled control (Figure 4b), which paralleled a significantly increased expression of the downstream effector thermogenic uncoupling protein 1, UCP1 (Figure 4c–e). Transfection with miR-337-3p inhibitor showed a significant reduction in UCP1 (Figure 4c–e). Moreover, there was a decreased protein abundance of the OXPHOS Complex V following modulation with miR-337-3p inhibitor (Figure 4d). Taken together, these findings demonstrate that the mitochondrial respiration is increased and favours thermogenesis, as evidenced by an increase in the uncoupling protein inducing brown fat metabolism.

### 3.5. miR-337/Twist1 Axis in Metabolic Syndrome Humans

**Figure 5:**
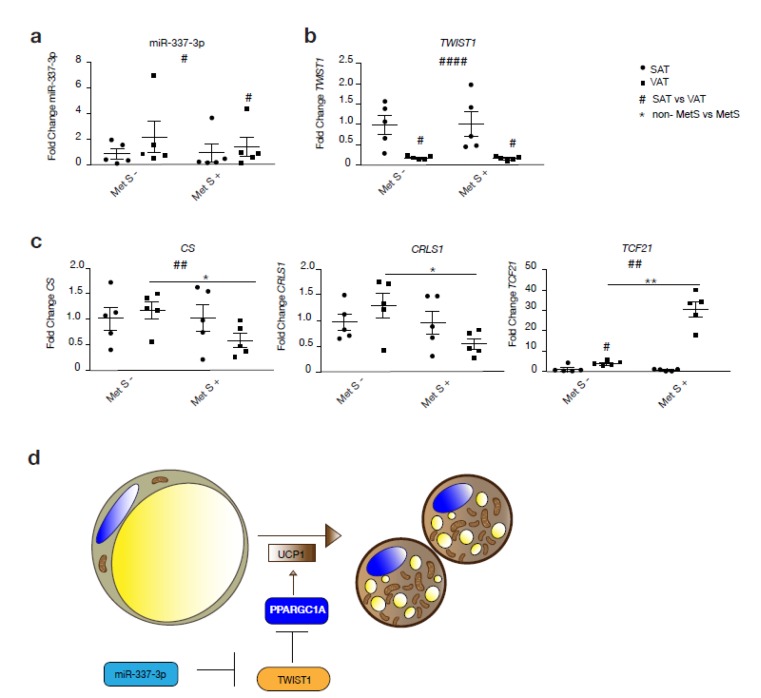
The miR-337-3p and Twist1 axis is disrupted in human subjects with MetS. (**a**) RT-PCR analysis of hsa-miR-337-3p in human VAT and SAT from non-MetS and MetS human subjects (n = 5/group). (**b**) RT-PCR analysis of *TWIST1* in human VAT and SAT from non-MetS and MetS human subjects (n = 5 per group). (**c**) RT-PCR analysis of *TCF21* and mitochondrial genes *CRLS1* and *CS* in VAT and SAT from non-MetS and MetS human subjects (n = 5 per group). (**d**) Illustration of the pathway proposed in the present study through which miR-337-3p induces browning of adipose tissue. TWIST1, when activated, inhibits PPARGC1A, which prevents the transcription of genes involved in brown fat metabolism. In summary, miR-337-3p induces translational inhibition of TWIST1 and prevents transcriptional inhibition of genes involved in the browning of adipose tissue. Data are presented as means ± SEM, and individual data points are given by the dot plot. *L7* mRNA was used as an internal normalizer to the number of mRNA transcript copies. 5S RNA was used as an internal normalizer to miRNA transcript copies. # *p* < 0.05; ## *p* < 0.01; #### *p* < 0.0001; Paired sample *t*-test (SAT vs. VAT) * *p* < 0.05; ** *p* < 0.01 Independent sample *t*-test (non-MetS vs. MetS), n, number of biological replicas; † VAT, visceral adipose tissue; SAT, subcutaneous adipose tissue; MetS, metabolic syndrome, TCF21, transcription factor 21; *CS,* citrate synthase; *CRLS1,* cardiolipin synthase 1;PPARGC1A, peroxisome proliferator-activated receptor-gamma coactivator-1 alpha; UCP1, uncoupling protein 1.

To assess whether the *TWIST1*-miR-337 axis is functional in humans, we assessed miR-337 and *TWIST1* gene expression levels in two forms of WAT: SAT and VAT, from subjects with or without MetS (Figure 5a–c). Correspondingly, we observed a significant increase in miR-337 expression in VAT inverse to the expression of *TWIST1* in VAT compared to SAT among MetS subjects (Figure 5a,b). Beside this, expression of miR-337-3p was generally increased in VAT compared to SAT, while expression of *TWIST1* was decreased in VAT compared to SAT irrespective of the presence or absence of MetS. Among non-MetS subjects, there was an increased expression of mitochondrial markers, such as *CRLS1* and *CS,* in VAT compared to SAT, whereas the opposite was observed among obese MetS individuals. Mitochondrial oxidative capacity of VAT from MetS subjects was significantly reduced by a decreased expression of *CS* and *CRLS1* compared to non-MetS VAT (Figure 5c). Moreover, the VAT in MetS individuals showed a stronger WAT profile by the expression of *TCF21*, a typical marker of WAT. Taken together, we found a positive correlation between miR-337-3p expression in human adipose tissue and serum high density lipoprotein (HDL) cholesterol levels (r = 0.715 *p* = 0.013).

## 4. Discussion

The present study demonstrates that miR-337-3p induced repression of Twist1, enhancing BAT specific gene expression and protein abundance in brown adipocytes. We demonstrated that miR-337-3p is highly expressed in BAT compared to WAT, and is increased during the differentiation of brown adipocytes. Although no previous associations have been made between miR-337-3p and browning of adipose tissue, a previous study has shown that this miR correlates with protection against saturated fatty acid-induced insulin resistance in mouse muscle cells [18]. Therefore, the described function of miR-337-3p in the present study could be a mediator of the contribution of BAT in glucose homeostasis and regulates insulin sensitivity [19].

We found that miR-337-3p exerts its effect by targeting Twist1. In line, the elevated expression of miR-337-3p in BAT compared to WAT was accompanied by a reduced Twist1 protein abundance in BAT compared to WAT, and was accompanied by increased expression of browning markers. Nairismägi analysed the presence of conserved miR target sites in the 3′UTR of the *Twist1* gene and identified for the first time miR-337-3p in combination with miR-151-5p, and miR-145a-5p and miR-151-5p as strong regulators of *Twist1* [20]. Heterozygous knockout mice of *Twist1* showed an obesity-resistant phenotype when placed on a high-fat diet and increased brown fat metabolism by elevated oxygen consumption, mitochondrial biogenesis, and uncoupling in BAT [16]. Recent studies have shown that overexpression of Twist1 is metabolically unfavourable as it indirectly contributes to fat accumulation, while the silencing of Twist1 enhances insulin sensitivity of adipocytes by antagonizing mitochondrial damage [21]. Moreover, in brown adipocytes, Twist1 interacts directly with Ppargc1a and serves as a key regulator of a negative feedback regulatory loop, orchestrating the balance between Ppargc1a induced transcription of target genes involved in uncoupling and Ppard isoform controlled brown fat metabolism resulting in a balanced energy homeostasis in response to energy substrate availability [16,22].

We performed a functional analysis that showed that overexpression of miR-337-3p leads to a decrease in TWIST1 and an increase in UCP1 protein abundance. UCP1 is a downstream effector present in the mitochondrial membrane and necessary for the brown fat specific thermogenic function. [23,24]. To confirm that there is an interaction between the miR and the 3′UTR of the target gene, reporter plasmids containing the 3′UTR of Twist1 were co-transfected with miR-337-3p, which validated the predicted seed complementarity between miR-337-3p and *Twist1*-3′UTR. Previously, Nairismägi et al. reported that miR-337-3p required the presence of miR-151-5p to induce translation inhibition of Twist1, although the data were not confirmed by Western blot analysis, but relied solely on luciferase reporter measurements [20]. Besides this, these experiments were performed on H1299 cells, a human non-small cell lung carcinoma cell line, while we performed our experiments in mouse brown adipocytes. In contrast to what this group has shown, in our model reverse transfection with miR-337-3p precursor was enough to induce translational inhibition not only validated by using reporter plasmids but also by direct measurement of TWIST1 protein abundance by Western blot analysis.

To date, obesity has reached large proportions worldwide, causing the development of high mortality comorbidities, such as diabetes, cardiovascular disease, and cancers. This underlines the dire need for novel treatment paradigms. In the present study, the human translatability and the potential for clinical application are substantiated by the validation of the inverse relationship between miR-337-3p and Twist1 shown in human adipose tissue samples. While miR-337-3p is increased, Twist1 is decreased in VAT compared with SAT. These findings are accompanied by an increase or a decrease in mitochondrial markers (CRLS1 and CS) in healthy subjects (however, not significant) and MetS obese subjects VAT, respectively. In physiological conditions, one would expect that following an increase in miR-337-3p and a decrease in Twist 1, an increase in the mitochondrial markers occurs. However, due to pathologic alterations attributed to MetS, the level of mitochondrial markers was decreased instead. The distribution of adipose tissue in obese individuals gives a tremendous amount of information on the cardiometabolic risk profile in humans [25]. In obesity, increased visceral adipose tissue distribution consistently confirms a strong association with metabolic complications and cardiovascular disease and malignancies [25,26,27,28]. The “unhealthy” profile of VAT in MetS obese subjects with a reduction in mitochondrial activity is in line with previous studies that reported impaired mitochondria activity in metabolic disorders, such as obesity and metabolic syndrome [29,30]. It seems that the effect of obesity and MetS in VAT is higher than the one produced by the effect by miR-337-3p increased, and Twist1 decreased. This deficit provides the possibility of a therapeutic window, aiming at counteracting mitochondrial dysfunction in adipose tissue from obese individuals. Although an important limitation of the present study is the small sample size of the human cohort. The results shown here for human data are in accordance with previous findings in literature. Particularly, CRLS1, a primary mediator in the activation and recruitment of thermogenic fat correlates positively with insulin sensitivity and could reduce insulin resistance in MetS [31]. Not only did miR-337-3p seem to have a beneficial effect on the expression of thermoregulatory and mitochondrial genes in adipose tissue, but this miR also correlated positively with serum levels of HDL cholesterol. HDL cholesterol is known for its beneficiary effects in reducing atherosclerotic plaques by reverse cholesterol transport [2]. These findings suggest multiple beneficiary effects possible by miR-337-3p. However, key to accomplishing these beneficiary effects and prior to clinical translation, future research should be directed towards the development of vehicles that allow delivery of the miR only to adipose tissue, to prevent off-target effects in other tissues that could potentially be harmful.

Conclusively, we here demonstrated that miR-337-3p targets Twist1, which is involved in the suppression of mitochondrial metabolism and function in brown adipocytes. Therefore, modulation of Twist1 by miR-337-3p represents a potential strategy to counteract obesity and its metabolic alterations. To date, previous studies have shown that subcutaneous WAT is able to take on many of the “visceral fat”-like molecular characteristics through miR-mediated signaling [32]. Moreover, human VAT is more prone to adopt a brown fat phenotype upon activation, raising interesting grounds for possible differentiation into mature brown fat [24,33]. Future studies could focus on different strategies for the delivery of this miR specifically to VAT to convert harmful VAT in beneficial energy combustion, to accomplish its beneficial effects.

## Figures and Tables

**Table 1:** Primer sequences.

Gene Name	Gene ID	Sequence	
**Primers for *Mus Musculus***			
*L7*	NM_011291	Forward	GAAGCTCATCTATGAGAAGGC
		Reverse	AAGACGAAGGAGCTGCAGAAC
*Twist1*	NM_011658	Forward	CAGGCCGGAGACCTAGATG
		Reverse	CCACGCCCTGATTCTTGTG
*Cs*	NM_026444	Forward	TAAGGAGCAGGCCAGAATTAAG
		Reverse	CCGAAGTCTCATACACAAGTCC
*Crls1*	NM_001024385	Forward	GCTCTTGATCCACTTGCTGATA
		Reverse	GTAAGTGAGTGGGACTGGAATAAG
*Ucp1*	NM_009463	Forward	GGGAGAGAAACACCTGCCTCT
		Reverse	GGGAGAGAAACACCTGCCTCT
*Cox8b*	NM_007751	Forward	TTGGGGCCAAGGAAGGAGTG
		Reverse	GAGATCCCCACAGCCTGCTC
miR-337-3p			TCAGCTCCTATATGATGCCTTT
**Primers for Humans**			
*TWIST1*	NM_000474	Forward	GCTCAGCTACGCCTTCTC
		Reverse	TGTCCATTTTCTCCTTCTCTGG
*CS*	NM_004077	Forward	GGCCATTGACTCTAACCTGG
		Reverse	CACTTACATTGCCACCCTCA
*CRLS1*	NM_019095	Forward	ATGACGAGAATTGGCTTGGC
		Reverse	TTTGATTGGCCCAGTTTCGA
*UCP1*	NM_021833	Forward	CGGAATCAAACCTCGCTACA
		Reverse	TGACACTTCTCATCAGATTGGG
*TCF21*	NM_003206	Forward	CAGATCCTGGCTAACGACAA
		Reverse	CGGTCACCACTTCTTTCAGG

## Supplementary Materials

The following are available online at https://www.mdpi.com/2073-4409/9/4/1056/s1, Table S1: Anthropometric and clinical characteristics of healthy and metabolic syndrome subjects: Table S2: Strategy for selecting gene of interest and literature search.

## Abbreviations

ACTB	(actin beta)
ATP	(adenosine triphosphate)
ATPB	(β-subunit of ATP synthase)
BAT	(brown adipose tissue)
Crls1	(cardiolipin synthase 1)
Cs	(Citrate synthase)
GAPDH	(glyceraldehyde- 3 Phosphate dehydrogenase)
miR	(micro-RNA)
MetS	(Metabolic syndrome)
OXPHOS	(oxidative phosphorylation)
*PPARGC1A*	(peroxisome proliferative activated receptor, gamma, co-activator alpha)
PPARD	(peroxisome proliferative activated receptor delta)
RT-PCR	(real-time polymerase chain reaction)
SAT	(subcutaneous adipose tissue)
TPS	(total protein stain)
Twist1	(twist family basic helix–loop–helix transcription factor 1)
CP1	(uncoupling protein1)
3′UTR	(3′ untranslated region)
VAT	(visceral adipose tissue)
WAT	(white adipose tissue)
