# Supplementary material for: MiR-337-3p Promotes Adipocyte Browning by Inhibiting TWIST1
Source: Cells. 2020 Apr 23;9(4):1056. doi: 10.3390/cells9041056 (PMC7226112; doi:10.3390/cells9041056)
Supplement: Supplementary file 1 [file cells-09-01056-s001.pdf]

cells

MDPI

*Supplementary Information*

# MiR-337-3p Promotes Adipocyte Browning by Inhibiting TWIST1

**Indira G.C. Vonhögen [1], Hamid el Azzouzi [1,2], Servé Olieslagers [1], Aliaksei Vasilevich [3], Jan de Boer [3], Francisco J. Tinahones [4,5], Paula A. da Costa Martins [1,6], Leon J. de Windt [1,*] and Mora Murri [4]**

1 Department of Molecular Genetics, Faculty of Sciences and Engineering; CARIM School for Cardiovascular Diseases, Faculty of Health, Medicine and Life Sciences, Maastricht University,6200 MD Maastricht, The Netherlands; indira.vonhogen@maastrichtuniversity.nl (I.G.C.V.); h.elazzouzi@erasmusmc.nl (H.e.A.); s.olieslagers@maastrichtuniversity.nl (S.O.); p.dacostamartins@maastrichtuniversity.nl (P.A.d.C.M.)
2 Department of Molecular Genetics, Erasmus University MC, 3015 GD Rotterdam, The Netherlands
3 Department of Biomedical Engineering and Institute for Complex Molecular Systems, Eindhoven University of Technology,5600 MB Eindhoven, The Netherlands; a.vasilevich@tue.nl (A.V.); j.d.boer@tue.nl (J.d.B.)
4 Unidad de Gestión Clínica de Endocrinología y Nutrición, Instituto de Investigación Biomédica de Málaga (IBIMA), Hospital Clínico Virgen de la Victoria, 29010 Málaga, Spain; CIBER Fisiopatología de la Obesidad y Nutrición (CIBEROBN), Instituto de Salud Carlos III, Málaga, Spain; fjtinahones@hotmail.com (F.J.T.); moramurri@gmail.com (M.M.)
5 Faculty of Medicine, University of Malaga, 29010 Malaga, Spain
6 Department of Physiology and Cardiothoracic Surgery, Faculty of Medicine, University of Porto, 4200-319 Porto, Portugal
* Correspondence: l.dewindt@maastrichtuniversity.nl

Received: 28 March 2020; Accepted: 15 April 2020; Published: date

## SUPPLEMENTARY TABLE

**Table S1.** Anthropometric and clinical characteristics of healthy and metabolic syndrome subjects.

| | Non-MetS | MetS | *p*-Value |
|---|---|---|---|
| **Variables** | **n=5** | **n=5** | |
| **Age** (years) | 53 ± 5 | 48 ± 2 | 0.320 |
| **BMI** (kg/m$^2$) | 22 ± 1 | 53 ± 4 | **<0.0001** |
| **Waist circumference** (cm) | 78± 2 | 150 ± 16 | **<0.0001** |
| **SBP** (mmHg) | 136 ± 4 | 133 ± 5 | 0.633 |
| **DBP** (mmHg) | 79 ± 6 | 79 ± 9 | 0.971 |
| **Triglyceride** (mmol/L) | 1.0 ± 0.1 | 1.7 ± 0.2 | **<0.05** |
| **HDL cholesterol** (mmol/L) | 1.8 ± 0.2 | 0.5 ± 0.1 | **< 0.05** |
| **Fasting glucose** (mmol/L) | 5.3 ± 0.1 | 6.2± 0.5 | 0.131 |
| **HbA1c** (%) | 5.2 ± 0.13 | 5.9 ± 0.3 | 0.103 |
| **HOMA-IR** | 1.1 ± 0.3 | 7.3 ± 2.2 | 0.067 |

Data are means ± SEM. Abbreviations: BMI, Body mass Index; DBP, diastolic blood pressure; SBP, systolic blood pressure; HDL, high density lipoprotein; HbA1c, glycosylated Hemoglobin A1C; HOMA- IR, homeostasis model assessment of insulin resistance index. All statistics were performed using Mann Whitney-U test.

**Table S2.** Strategy used for selecting gene of interest and literature search. Genes and corresponding publications listed below are investigated with the main aim of identifying genes of interest for studying browning. Next by inserting the selected gene miRNA's that target gene of interest are investigated in miRNAviewer Mouse and Rat databases. This led to the identification of Twist-1. The miRNAviewer output helped with composing the list of miRNAs that were assessed in BAT and WAT.

| Gene | mirnaviewer Mouse Transcript | mirnaviewer Rat Transcript | Published |
|---|---|---|---|
| **Aldh1a1** | | | **miR-155** http://www.ncbi.nlm.nih.gov/pubmed/26317551 |
| **Atg7** | 79:miR-24 | | **miR-210 miR-20a MIR137 miR-199a-5p miR-375** |
| **Atf4** | | | **miR-214** Free Radic Biol Med. 2016 Mar;92:39-49. RNA Biol. 2015;12(3):343-53. J Biol Chem. 2015 Mar 27;290(13):8185-95. Nat Med. 2013 Jan;19(1):93-100. |
| **Eif4ebp1** | 1:**miR-138**, 128:**miR-125b**, 163:**miR-138**, 170:**miR-202**, 390:**miR-135a**, 391:**miR-135b** | 1:miR-138, 127:miR-125a, 128:miR-125b, 134:miR-18, 135:miR-18, 170:miR-202, 390:miR-135a, 391:miR-135b | **miR-125a and miR-125b** Oncotarget. 2016 Feb 23;7(8):8726-42. **miR-9-5p, miR-675-5p, and miR-138-5p** Int J Mol Sci. 2016 Feb; 17(2): 236. **miR-34c-3p** Cell Prolif. 2015 Oct;48(5):582-92. miR-768-3p Oncogene. 2014 May 15;33(20):2577-88 |
| **Eif4ebp2** | 128:miR-96, 1112:miR-330, 1123:miR-214, 1572:miR-145, 2016:miR-125b, 2017:miR-125a | 32:miR-18, 128:miR-96, 436:miR-185, 792:miR-324-3p, 1112:miR-330, 1183:miR-214, 1209:let-7b, 1229:miR-185, 1833:miR-331 | |
| **Id1** | 250:miR-326 | 250:miR-326 | **miR-381** J Thorac Oncol. 2012 Jul;7(7):1069-77. **miR-29b** Oncogene. 2012 Sep 20;31(38):4221-32. |
| **Ikbke** | 92:let-7c, 92:let-7b, 92:let-7f, 92:let-7a, 93:let-7e, 93:miR-98, 94:let-7g, 96:let-7i, 99:let-7d | 92:let-7c, 92:let-7b, 92:let-7f, 92:let-7a, 93:let-7e, 93:miR-98, 94:let-7g, 96:let-7i, 99:let-7d, 150:miR-324-5p | miR-155-5p Oncotarget. 2016 Mar 29;7(13):16567-80. Neoplasia. 2009 Feb;11(2):167-76 **let-7e-5p, miR-98-5p and miR-145a-5p** Biochim Biophys Acta. 2015 Aug;1852(8):1585-98 Hsa-miR-4717-5p Am J Med Genet B Neuropsychiatr Genet. 2015 Jun;168B(4):296-306 **let-7b and let-7i** Biochem Biophys Res Commun. 2015 Mar 6;458(2):307-12. **miR-296** RNA. 2012 Jan;18(1):135-44. |
| **Mstn** | 550:miR-29a | 550:miR-29a | **miR-27** Biomed Res Int. 2015;2015:391306. Am J Physiol Cell Physiol. 2011 Jan;300(1):C124-37. PLoS One. 2014 Jan 31;9(1):e87687. BMC Genomics. 2013 Mar 19;14:194. **miR-128** Cell Signal. 2015 Sep;27(9):1895-904. |
| **Prlr** | 35:**miR-142-3p**, 135:**miR-185**, 426:**miR-122a**, 499:**miR-30a-5p**, 503:**miR-30e**, 840:**miR-124a** | 1:**miR-138**, 127:**miR-125a**, 128:**miR-125b**, 134:**miR-18**, 135:**miR-18**, 170:**miR-202**, 390:**miR-135a**, 391:**miR-135b** | miR-135a DNA Cell Biol. 2015 Aug;34(8):534-40. |
| **p107** | 12:miR-302b, 12:miR-302, 12:miR-20, 465:miR-122a | 12:**miR-302b**, 12:**miR-302** | **miR-888** Cell Cycle. 2014;13(2):227-39 **MiR-17, MiR-20a, and MiR-106b** PLoS One. 2011 Jan 20;6(1):e16138. **miR-106b-5p**' Oncogene. 2014 Oct 2;33(40):4813-22. |

| | | | |
|---|---|---|---|
| **Smad3** | 113:miR-324-5p, 153:miR-326, 278:miR-138, 1184:miR-204, 1184:miR-211, 1477:miR-15b, 1617:miR-23b, 1780:miR-342, 1781:miR-377, 2827:miR-18, 2849:miR-18, 3101:miR-346, 3562:miR-377, 3653:miR-377, 4071:miR-185, 4336:miR-15a, 4542:miR-302b*, 4607:miR-28 | 855:miR-199a, 855:miR-199b, 1576:miR-412, 3903:miR-145 | **MiR-23a-3**p Biochem Biophys Res Commun. 2016 Jun 16. miR-23b PLoS One. 2015 Jul 8;10(7):e0131867. miR-145 and miR-203 Lung Cancer. 2016 Jul;97:87-94. Cell Mol Biol (Noisy-le-grand). 2015 May 8;61(3):12-6. Biochem Biophys Res Commun. 2015 Mar 27;459(1):49-53. **MiR-195** Int J Gynecol Cancer. 2016 Jun;26(5):817-24 miR-16-5p Curr Pharm Des. 2015;21(35):5160-7. miR-199a-5p Biochem Biophys Res Commun. 2016 May 13;473(4):859-66 miR-21 Clin Sci (Lond). 2015 Dec;129(12):1237-49. miR-323-3p Oncotarget. 2016 Mar 22;7(12):14912-24. miR-590-5p Cell J. 2016 Spring;18(1):7-12 miR-708 Sci Rep. 2016 Mar 2;6:22599.MiR-23a-3p Biochem Biophys Res Commun. 2016 Jun 16. **miR-23b** PLoS One. 2015 Jul 8;10(7):e0131867. miR-145 and miR-203 Lung Cancer. 2016 Jul;97:87-94. Cell Mol Biol (Noisy-le-grand). 2015 May 8;61(3):12-6. Biochem Biophys Res Commun. 2015 Mar 27;459(1):49-53. MiR-195 Int J Gynecol Cancer. 2016 Jun;26(5):817-24 miR-16-5p Curr Pharm Des. 2015;21(35):5160-7. miR-199a-5p Biochem Biophys Res Commun. 2016 May 13;473(4):859-66 miR-21 Clin Sci (Lond). 2015 Dec;129(12):1237-49. miR-323-3p Oncotarget. 2016 Mar 22;7(12):14912-24. miR-590-5p Cell J. 2016 Spring;18(1):7-12 miR-708 Sci Rep. 2016 Mar 2;6:22599. |
| **Tnfrsf1a** | 105:miR-29b, 106:miR-29a, 270:miR-138 | | **miR-29a** World J Gastroenterol. 2016 May 28;22(20):4881-90. miR-511 EMBO Mol Med. 2015 May 20;7(8):1004-17. |
| **Trpv4** | 1:miR-17-3p | 1:miR-17-3p | **miR-203** Arch Oral Biol. 2013 Feb;58(2):192-9. |
| **Twist1** | 57:miR-151, 68:miR-199a, 68:miR-199b, 69:miR-337, 253:miR-361, 447:miR-199b, 461:miR-145, 600:miR-220 420:let-7c, 420:let-7b, 597:miR-151, 608:miR-199b, 608:miR-199a, 609:miR-337, 793:miR-361 | 14:miR-370, 57:miR-151, 68:miR-199a, 68:miR-199b, 69:miR-337, 253:miR-361, 461:miR-145, 600:miR-220 15:miR-370, 72:miR-371, 420:let-7b, 597:miR-151, 608:miR-199b, 608:miR-199a, 609:miR-337, 793:miR-361 | mir-186 Oncotarget. 2016 Apr 21. Oncogene. 2016 Jan 21;35(3):323-32. miR-23a Oncol Rep. 2015 Feb;33(2):942-50. miR-26b-5p Oncotarget. 2016 Mar 24. miR-1271 Biochem Biophys Res Commun. 2016 Apr 1;472(2):346-52 MiR-548c J Exp Clin Cancer Res. ...2016 Jan 13;35:10. miR-33b Sci Rep. 2015 Apr 28;5:9995. MiR-520d-5p Oncotarget. 2014 Dec 15;5(23):12141-50.... |
